# Simulating Peptide Monolayer Formation: GnRH-I on Silica

**DOI:** 10.3390/ijms22115523

**Published:** 2021-05-24

**Authors:** Neret Pujol-Navarro, Karina Kubiak-Ossowska, Valerie Ferro, Paul Mulheran

**Affiliations:** 1 Department of Chemical and Process Engineering, University of Strathclyde, 75 Montrose Street, Glasgow G1 1XJ, UK; paul.mulheran@strath.ac.uk; 2 Strathclyde Institute of Pharmacy and Biomedical Sciences, University of Strathclyde, 161 Cathedral Street, Glasgow G4 0RE, UK; v.a.ferro@strath.ac.uk; 3 ARCHIE-WeSt, Department of Physics, University of Strathclyde, 107 Rottenrow East, Glasgow G4 0NG, UK; karina.kubiak@strath.ac.uk

**Keywords:** GnRH-I, molecular dynamics, adsorption

## Abstract

Molecular dynamics (MD) simulations can provide a detailed view of molecule behaviour at an atomic level, which can be useful when attempting to interpret experiments or design new systems. The decapeptide gonadotrophin-releasing hormone I (GnRH-I) is known to control fertility in mammals for both sexes. It was previously shown that inoculation with silica nanoparticles (SiNPs) coated with GnRH-I makes an effective anti-fertility vaccine due to how the peptide adsorbs to the nanoparticle and is presented to the immune system. In this paper, we develop and employ a protocol to simulate the development of a GnRH-I peptide adlayer by allowing peptides to diffuse and adsorb in a staged series of trajectories. The peptides start the simulation in an immobile state in solution above the model silica surface, and are then released sequentially. This facile approach allows the adlayer to develop in a natural manner and appears to be quite versatile. We find that the GnRH-I adlayer tends to be sparse, with electrostatics dominating the interactions. The peptides are collapsed to the surface and are seemingly free to interact with additional solutes, supporting the interpretations of the GNRH-I/SiNP vaccine system.

## 1. Introduction

Molecular dynamics (MD) simulations provide a detailed analysis at an atomistic level of molecule interactions with the environment and other molecules and materials. Simulation systems can be designed to study the effect of different physiological conditions such as pH, ionicity, and temperature on molecular behaviour over time. Consequently, the simulations can be used to design effective technologies with optimal performance. For example, MD simulations can be used to study many biomedical-related issues such as drug resistance [1], drug development [2], effects of mutations in molecules [3], protein folding [4], interactions between molecules [5], protein binding [6], protein adsorption and interactions with both organic and non-organic surfaces [7], detailed studies of enzymatic activity [8], drug delivery [9] and the oligomerisation process [10], among others.

MD simulations offer various advantages over the use of wet laboratory experiments, representing an attractive method for research. Aside from the ability to visualise biological processes at a molecular level, MD simulations are accessible to most scientists, as many simulations can now be performed locally using the graphics processing units (GPUs) present in most modern computers at a reasonable cost [11,12,13]. Moreover, technological advances have increased the speed of the simulations, with standard computers outperforming the speeds previously only achievable on most supercomputers [11]. As a result, simulations have become fast and relatively inexpensive research methods that can aid scientists to obtain insightful, reliable, fast, and inexpensive data.

Gonadotrophin-releasing hormone I (GnRH-I) is a 1.18 kDa decapeptide with the sequence Glu-His-Trp-Ser-Tyr-Gly-Leu-Arg-Pro-Gly. It is neutral at pH 7, but has the charged residues glutamic acid (Glu, negative) and arginine (Arg, positive). It is primarily hydrophilic, with the exception of leucine (Leu) in the seventh position. It is produced by the hypothalamus that stimulates the release of follicle-stimulating hormone (FSH) and luteinising hormone (LH) in females and males, regulating fertility [14].

One approach to controlling fertility in both male and female mammals is the use of immunisation against GnRH-I [15,16,17,18]. This induces the production of GnRH neutralising antibodies that bind to endogenous circulating GnRH-I, preventing it from stimulating FSH and LH secretion [19]. An established anti-GnRH vaccine, GonaCon, has been employed successfully in the control of wild animal populations [20]. Moreover, GnRH-I has been observed to be expressed in approximately 80% of human ovarian and endometrial cancers and in 50% of breast cancers [21], as well as in prostate and bladder cancers [22], potentially enabling neutralising antibodies to interact with these cancers. In order to raise these antibodies, since GnRH-I is a naturally occurring self-peptide, it must be conjugated to a foreign carrier or adjuvant (such as silica nanoparticles (SiNPs)) to ensure the stimulation of the required immune response [23].

Work performed by the authors’ group [24] assessed the in vivo effects of GnRH-I-SiNP conjugates on mouse antibody production and testosterone levels. Results showed that antibody response was low, at close to baseline (*p* < 0.025). However, testosterone levels were very low, with a >95% decrease (*p* < 0.001). These results were interpreted using MD simulations of the adsorption of GnRH-I to the SiNPs. These showed that the adsorbed peptide is collapsed in nature, and therefore less available to interact with antigen-presenting cells—hence, the low antibody response to the GnRH-I-SiNP conjugates. Nevertheless, the terminal residues of the adsorbed peptide are observed to be free to interact with the environment. These play a key role in the peptide interactions with the GnRH receptor, so that the conjugates can be effective at blocking the receptors. This explains the resulting low antibody levels but high testosterone suppression [24].

In this work we extend the simulation study of GnRH-I adsorption onto silica. In the experiments, the SiNPs are loaded with multiple peptides, so that behaviour in the crowded monolayer environment can be assessed. This was briefly attempted in the previous work by simulating an island of peptides on the silica surface [24]. However, this did not faithfully follow the assembly of the monolayer by sequential adsorption of peptide from the solution. Therefore, we have designed a new methodology for simulating the layer formation, one that might easily be applied to other adsorbing peptide systems, and use it for our current model system. This allows us to consider the monolayer formation process, and to revisit our interpretations of the in vivo experiments.

The paper is structured as follows. First, we present the results on the monolayer formation process, followed by an analysis of the GnRH-I configurations. We follow with the methods used to simulate our model GnRH-I peptides adsorbing to the silica surface, and finish with a summary and the conclusions drawn from this study.

## 2. Results and Discussion

### 2.1. Peptide Layer Formation

**Figure 1:**
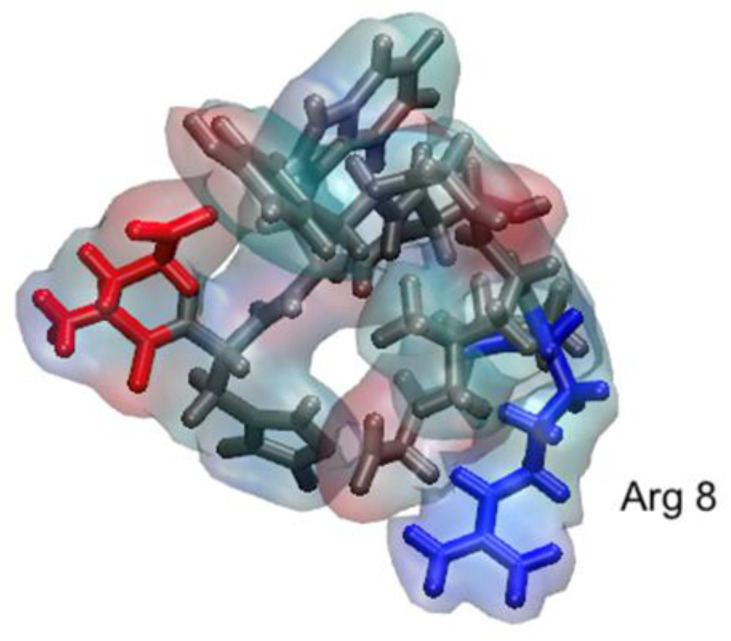
Simulated GnRH-I structure in solution using a transparent surface overlapped on the liquorice representation in VMD. Positive side chains are shown in blue, negative in red, and neutral in grey, and Arg8 is annotated.

**Figure 2:**
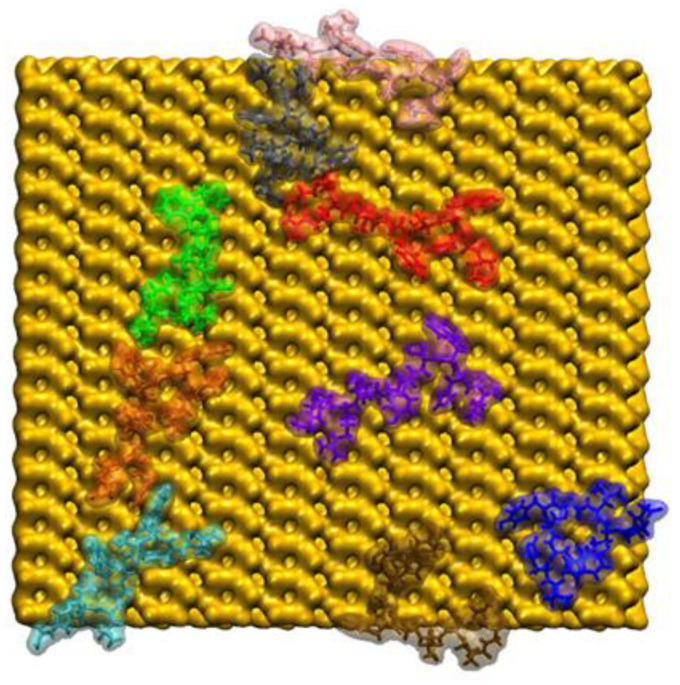
The final configuration of the 9 GnRH-I peptides adsorbed to the model silica surface as viewed from above. The GnRH-I proteins are shown as transparent surfaces overlapped on opaque licorice VMD representations with each peptide given a different colour for ease of identification (see Table 1), while the silica as a yellow surface. The water molecules and ions are not shown for clarity.

The initial structure of the GnRH-I peptide is shown on Figure 1 while the arrangement of the nine adsorbed peptides at the end of the simulation is displayed in Figure 2.

**Figure 3:**
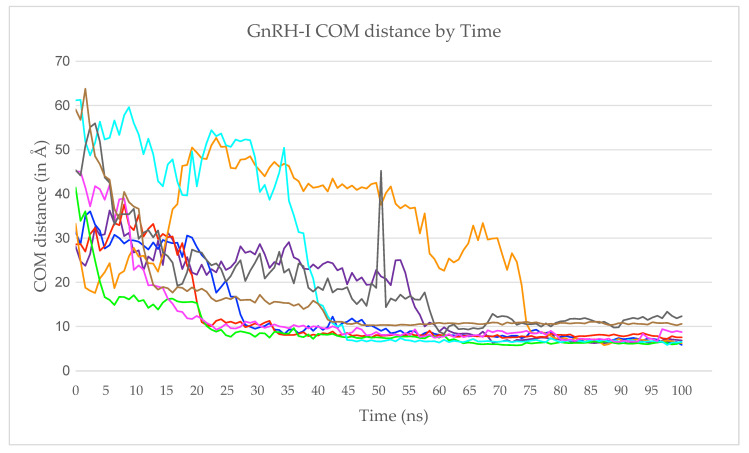
The GnRH-I centre-of-mass (COM) z-coordinates measured for a period of 100 ns following their release. The trace colours match those given in Table 1.

**Figure 4:**
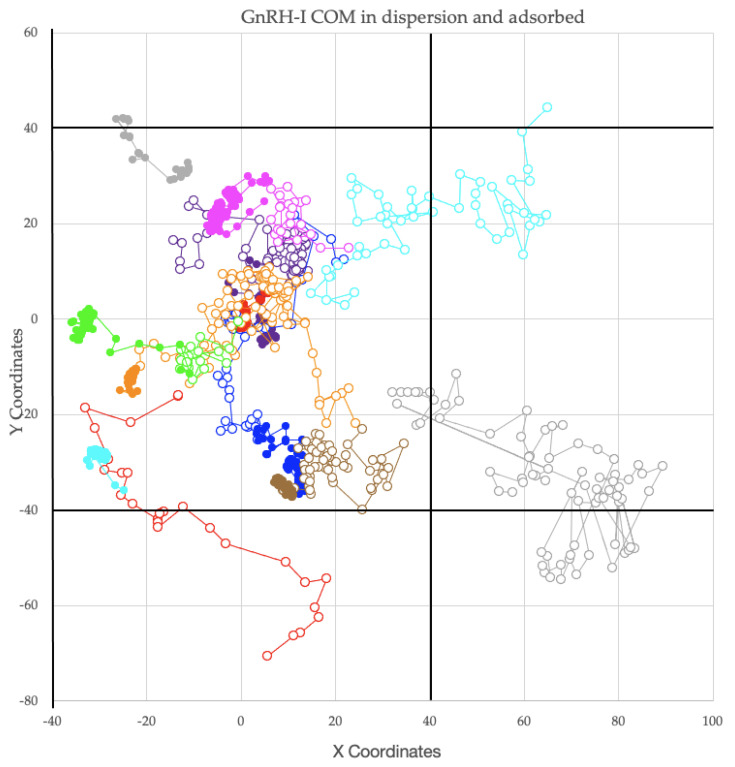
Peptide COM (x, y) coordinate traces measured in Å. The traces are shown as continuous lines utilizing the periodic boundary conditions. Each peptide’s trace is coloured as in Table 1. Empty circles represent peptides before adsorption, solid circles after. The simulation cell is indicated with black solid lines, also indicating the periodic boundary conditions in the (x, y) directions.

**Figure 5:**
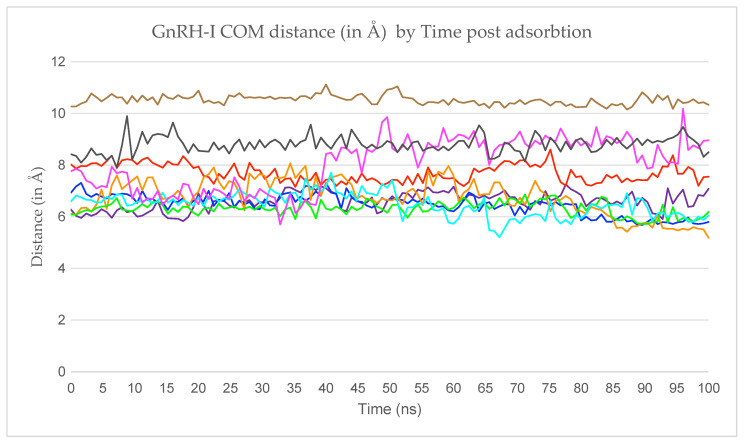
GnRH-I COM distance from the silica surface as a function time, starting from when all 9 peptides were adsorbed. The peptide traces are coloured as in Table 1.

**Figure 6:**
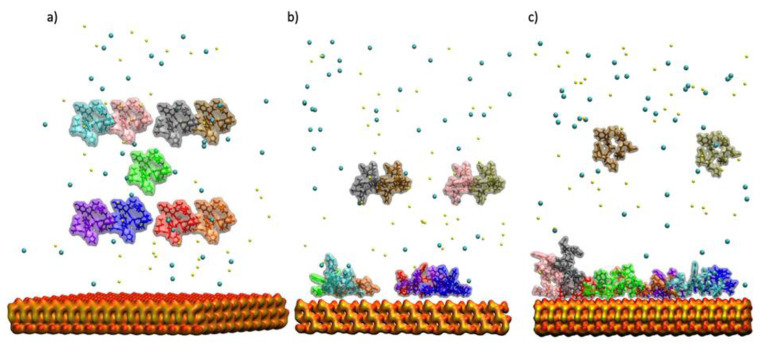
The simulation cell illustrating the GnRH-I starting positions above the model silica surface. (**a**) The initial 9 peptides; (**b**) the second simulation with 4 more peptides; (**c**) the third simulation with a further 2 peptides. GnRH-I proteins are displayed using a transparent surface overlapped on the VMD ‘liquorice’ representation, with each peptide given a different colour for ease of identification (see Table 1). Ions are shown as VdW spheres, Na^+^ ions are yellow while Cl^−^ ions are blue. The silica slab is represented by a surface with red oxygen and yellow silicon atoms. The water is not shown for clarity.

As explained in the Section 3, the peptides were released sequentially and allowed to adsorb one-by-one to the model silica surface; each peptide diffuses for 50 ns before the next peptide is released. For convenience, the peptides have been coloured to identify them as listed in Table 1. They are numbered by the order in which they were released, and it is clear from Figure 2 that there is no preference for subsequent peptides to adsorb next to previously adsorbed ones, nor indeed for multilayer formation. The colours in Table 1 will also be used to identify the peptides in Figures 3–6, where some quantitative data are presented.

All the peptides are in direct contact with silica surface, and most peptides interact with their neighbours. However, it is clear that the peptides tend to spread out on the surface, rather than cluster together. This is similar to the behaviour observed in earlier work where a dense island of the peptide, starting as a close-packed square array on the surface, was seen to spread out over a 50 ns trajectory [24]. One reason for this behaviour is that the GnRH-I interacts strongly with the silica through its N-terminus and positively charged Arg8 residue (Figure 1); this is explored in more detail below.

The image shows that the surface is not saturated with peptide, yet in the trajectories, 6 of the 15 possible peptides have not adsorbed to the surface despite starting closer to it than to the silicon-rich image surface (see Methods below). One explanation for this is the need for charge compensation at the surface. The electric field above the silica (which is negatively charged at pH 7) is screened by the Na^+^ ions. The adsorbing peptide disrupts the layer of ions, which must re-arrange to accommodate them, and since the peptides here are neutral the ions stay close to the surface rather than move to the bulk solution. This squeezing of the space available for the ions might be responsible for our inability to adsorb further peptides. The important role played by the screening ions has been identified in protein-adsorption simulations, notably when adsorbing the negatively charged BSA to the negatively charged silica surface [7].

In Figure 3 we trace the z-coordinate of each peptide’s centre-of-mass (COM) over time, where z is the axis normal to the silica surface and the oxygen atoms of the surface all have coordinate z = 0. Here, time is measured from the moment each peptide is released from its frozen state, rather than the start of the entire simulation. This means that we can readily assess how long it takes each peptide to adsorb.

Peptides 2, 3, 5, and 7 adsorbed rapidly to the silica surface (<30 ns), peptides 6 and 9 adsorbed at an intermediate rate (30–50 ns), and peptides 1, 4, and 8 took longer still (>50 ns) (Figure 3). The reason for the late adsorption of peptide 1 is that when first released it interacted with the still-frozen peptide 2 for around 50 ns. This interaction occurred between the carboxyl-terminus of Gly10 in peptide 1 and the hydrogens in the Arg8 side chain from peptide 2, implying that there was a strong electrostatic component to the attraction. We note, of course, that this interaction is an unwelcome artefact of the simulation methodology. Peptide–peptide interactions might occur in solution, but both should be fully mobile if the simulation is to be realistic. Indeed, this strong interaction was broken a few nanoseconds after peptide 2 was released from its frozen state; however, peptide 1 adsorbed to the silica surface while still interacting with peptide 2, with both being mobile and therefore representing a more realistic adsorption event. The peptide 1 COM was kept at around 18 Å from the silica surface as it was simultaneously being pulled up by peptide 2 and down by the attraction towards the silica surface. Once the interactions with peptide 2 ceased, peptide 1’s COM became drastically closer to the silica surface, where it remained strongly adsorbed. At the same time, peptide 2 was then able to adsorb rapidly. Referring back to Figure 2, we see that the two peptides ended up well separated on the surface, showing the short-range attraction to the surface outweighs inter-peptide attractions on the sparsely populated surface (peptides 1 and 2 were the first to adsorb).

Peptide 4’s adsorption process was similar to that of peptide 1. When released into the solution, peptide 4 diffused closer to the silica surface, but after around 15 ns, the aromatic ring of Tyr5 interacted with the aromatic ring of His2 of the frozen peptide 5. Peptide 4 then entwined with peptide 5, interacting through other residues, and only began to interact with the silica surface once peptide 5 was released, when it adsorbs to the silica surface almost immediately. Again, this shows that although the peptides might interact in solution, once a small cluster of peptides adsorbs to the silica surface, the peptides will preferentially interact with the surface. Referring again to Figure 2, peptide 4 (orange) is next to peptide 5 (green), but they both are collapsed to the surface rather than interacting strongly with each other.

In summary, both peptide 1 and peptide 4 interacted with frozen peptides (peptides 2 and 5 respectively), halting the adsorption process. The adsorption process was resumed after the frozen peptide was released into the simulation, with peptides 1 and 4 then adsorbing quickly to the silica surface.

Figure 4 shows traces of the peptides’ COMs in the (x, y) plane across the silica surface (all distances are measured in Å). Each trace is taken from 100 ns of the trajectory following the peptide’s release. For this reason, the adsorbed protein COMs do not correspond to the positions shown in Figure 2, which is from the end of the simulation. The empty circles represent data coordinates from diffusing peptides prior to adsorption, while solid circles represent data coordinates from adsorbed peptides (the colour scheme form Table 1 is used). It is clear that peptides 2, 3, 4, 6, and 8 diffused laterally above the silica surface before adsorbing, while peptides 1, 5, 7, and 9 adsorbed very close to their initial position above the surface. However, this diffusion does not seem to be correlated with adsorption speed, as, for example, peptides 2 and 3 adsorbed rapidly to the silica surface, but also diffused laterally before adsorbing. 

Once adsorbed, only peptide 2, 5, and 8 COMs showed significant movement across the surface. The other peptides showed small lateral COM movements caused by configurational flexing without long-range motion. This does not mean that they cannot diffuse across the surface, just that they are immobile on the 100 ns timescale of these atomistic trajectories. It is possible to probe longer timescale lateral diffusion using Steered MD [25].

Peptide 5’s COM moved across the surface post-adsorption as the peptide stabilised. When it first adsorbed, the first two residues of the protein, Glu and His, did not interact with the surface. Once the protein changed position and made contact with the surface via the positively charged N-terminus, the peptide’s COM became stable throughout the remaining simulation. This shows that while the forces between GnRH-I and the silica surface are strong, peptides can be quite mobile until they find a stable configuration. 

When peptide 8 first adsorbed to the surface, it interacted with peptide 7. At first, the Gly10 side chain in peptide 8 interacted with the side chain of arginine 8 from peptide 7. Peptide 8 then moved over peptide 7 and re-adsorbed to the silica surface between peptide 7 and peptide 3. This indicates why multilayer adsorption is not found in these simulations; peptides can be quite mobile, moving around one another, but become anchored to the surface once they have found a stable configuration. As will be seen below, there are some prominent low energy configurations that GnRH-I peptides can achieve.

### 2.2. GnRH-I Adsorption Configurations

The adsorbed GnRH-I COM distance from the silica surface is shown in Figure 5 as a function of time, starting from when each peptide has successfully adsorbed to the surface. The mean distance, range, and standard deviation values for each peptide can be found in Table 2. The results show that most peptide COMs were 6.50–7.50 Å away from the silica surface, indicating a fairly uniform monolayer with consistent peptide configurations throughout. The extreme cases are worth further consideration: peptides 7, 8, and 9 have the largest mean distance, and peptides 4 and 7 have the largest ranges (and standard deviations) in their COM distances from the surface. 

Peptide 7’s COM distance from the silica surface was the third largest of all adsorbed GnRH-I peptides in the simulation, at 8.05 Å, due to its interaction with peptide 3. As they interacted, peptide 7 became more stretched normal to the surface plane, causing its COM distance from the surface to increase. As Table 2 shows, peptide 7’s range was also the highest, as it experienced the largest change in COM distance from the surface throughout the simulation. This suggests that inter-peptide interaction can cause large changes in the COM distance from the silica surface post adsorption. 

Peptide 8 shows the second largest COM distance of all adsorbed peptides. This is due to its interaction with peptides 7 and 3, as mentioned previously. Peptide 8 crawled over peptide 7 to fully adsorb and then remained stable in between peptides 3 and 7. The increase in COM distance from the surface observed in the first third of Figure 5 is due to the “crawling”.

Peptide 9′s COM distance from the silica surface was the largest; however, it had the smallest distance range of all peptides, showing that although it was further away from the surface compared to the other peptides, the interaction was strong and stable. The large COM distance is due to the position of peptide 9′s amino acids and how it adsorbed to the surface. No ions were found to be trapped in between the silica surface and peptide 9, which could have been responsible for the relatively large COM. 

In summary, the large COM distance from the silica surface of peptides 7, 8, and 9 can be attributed to (a) the interactions of peptide 7 and 8 throughout the simulation; and (b) the peptide 9 amino acids, which are in contact with the silica surface as shown in Table 3. We now look in more detail at which residue side chains interact with the surface. 

Table 3 indicates the amino acids in contact with the silica surface for each peptide in its adsorbed state. Most GnRH-I adsorbed in a similar way, with Glu, His, Tyr, Gly, Leu, and Arg commonly being in contact with the silica surface. However, peptides 3, 7, and 8 each had only three amino acids in contact with the surface. Moreover, Arg8 was the only amino acid consistently in contact with the surface, while Pro9 and Gly10 were consistently exposed to solution in all peptides with exception of peptide 9. This difference in adsorbed configuration is responsible for peptide 9′s high COM distance from the surface. 

The dominant adsorption configuration of the peptides can be understood in terms of electrostatics. The arginine sidechain has (at pH 7) a positively charged amine end-group that interacts strongly with the negatively charge oxygen of the silica surface. Similarly, the N-terminus has a positive amine group that can also interacting strongly with the surface oxygen. The glutamic acid sidechain at position 1, which carries a negatively charged carboxylic acid group, is then left exposed to the solution and away from the surface. It is interesting to observe that the glutamic acid residue interacts with the surface in this way if the neighbouring histidine, which is neutral at pH 7, also interacts with the surface. At the other terminus, the neutral glycine will stay desorbed due to the repulsion of the negatively charged C-terminus.

Further insight into the electrostatic interactions in the system can be gained from analysis of the dipole moment orientation on each peptide with respect to the surface plane. The dipole moment of each peptide was calculated at different points within their trajectory, as reported in Table 4. Here, the initial dipole moments were always the same as all GnRH-I peptides were positioned in the simulation cell with the same starting orientation. 

The time at which each peptide was adsorbed was used to obtain the data indicated in columns D1 and D2 in Table 4, which were taken from 33% and 66% of their trajectory in solution prior to adsorption. For example, in a protein that took 50 ns to adsorb to the silica surface, the dipole moments in solution that are reported were taken at 17 ns (D1) and 33 ns (D2) following the peptide release. Dipole moments of the peptides at the time of their adsorption, and at 10 and 20 ns following this, are also reported to assess any changes after adsorption. 

The results show that all the adsorbed peptides tend to have their dipole moment oriented towards the surface, antiparallel to the surface normal (the *z* axis). This is as expected for the alignment of dipoles in the electric field above the negatively charged silica surface. Of course, the peptides are not rigid; nevertheless, the propensity for the positively charged groups to be drawn towards the surface and the negatively charged away from it is clear. In fact, the peptides tend to increase their dipole moments as the approach the surface, becoming more elongated in response to the surface electric field. 

Peptide 8 provides an interesting contrast, with its final dipole moment being parallel to the surface and low in magnitude; this is most likely due to the fact that its N-terminus did not adsorb, at least on the timescale of the simulation. Peptide 5 also shows interesting behaviour, having a dipole moment directed away from the surface at the time of adsorption before re-orienting by 20 ns post-adsorption. As discussed above, when peptide 5 first adsorbs, its N-terminus does not interact with the surface, so that it is mobile across the surface until it finds a more stable orientation. It is therefore possible that the longer-term fate of peptide 8 is also to re-orientate and direct its dipole moment towards the surface as the other peptides do.

## 3. Methods

The computational simulations used in this work follow the methodology outlined in our previous work [26]. NAMD v. 2.12 [27] was employed, with the CHARMM27 force field and the TIP3P water model. We employed periodic boundary conditions, particle mesh Ewald for the electrostatic interactions [28], and a cut-off of 12 Å for short-range forces. To reduce the computational time, all bonds and angles in the water molecules were constrained, employing the SHAKE algorithm. Due to the screening effect of the ions present in the simulation, we did not apply any dipole corrections to the Ewald summation. Minimisation and heating used a 1.0 fs timestep, which was increased to 2.0 fs for the trajectory performed at a temperature of 310K controlled by Langevin thermostat (NVT ensemble). The computations were performed on the ARCHIE-WeSt high-performance computer (archie-west.ac.uk, UK).

Analyses of the trajectories were performed using VMD v. 1.9. [29]. The model silica surface was created as described in our previous work [24], modelling the surface chemistry observed experimentally [30], with ca. 1 siloxide group per nm^2^. Furthermore, the use of periodic boundary conditions with such a slab creates an electric field of 0.2 V Å^−1^ above the surface [26], again in line with experimental observations for the surface charge on Stöber SiNPs at pH 7. We previously modelled the partial deprotonation of the oxygen-rich surface in a study of the adsorption of spontaneous membrane-translocating peptides [31], reducing the electric field without producing significant new insights regarding the dominant role of electrostatics. Thus, here we employed the fully deprotonated surface as our model substrate with the experimentally relevant electric field. This electric field is screened by the addition of NaCl ions to the water; here, we used a silica slab of dimensions 86 Å × 80 Å × 13 Å with 44 Na^+^ ions to screen the electric field above the siloxide-rich surface, and 44 Cl^−^ ions to screen the positively charged Si-rich surface on the opposite side of the slab.

The native GnRH-I structure used was 1YY1.pdb [32] (see Figure 1), which is the full protein of interest here. As a decapeptide, it has a flexible random coil structure with a single bend. The residue protonation was taken to be that at pH 7. The initial adsorption simulations were prepared with 9 GnRH-I peptides at least 20 Å above the target siloxide-rich surface and at least 12 Å from each other, plus a distribution of the Na^+^ and Cl^−^ ions, and the system was solvated in a water box that extended at least 15 Å away from any peptide atom. The resulting systems contained ~80,000 atoms (Figure 6a). 

The simulation was divided into several stages. At each stage, 1 of the 9 starting peptides was released, with the remaining ones staying frozen in place above the surface. The simulation cell then underwent energy minimisation and equilibration, and then simulation for 50 ns under the NVT ensemble at 310 K. During this time, all previously released peptides were free to diffuse and adsorb to the surfaces (either the oxygen-rich surface of experimental interest, or the silicon-rich surface at the bottom of the slab which is available due to the periodic boundary conditions). Adsorbed peptides could interact with one another on the surfaces. In this way, we attempted to simulate the growth of the monolayer in a computationally straight-forward manner.

After the initial 9 peptides had been released and simulated (equivalent to 450 ns of NVT trajectory), a further set of 4 frozen GnRH-I peptides were added to the simulation cell at least 20 Å above the silica surface and at least 12 Å from each other (Figure 6b). The configuration of the peptides adsorbed to the oxygen-rich surface was kept from the previous trajectory; any peptides adsorbed to the silicon-rich surface were removed. The newly constructed simulation cell was then rehydrated, and the ions added. From here, the previous cycle of trajectories was re-started, sequentially releasing the new peptides and simulating for 50 ns with all the mobile peptides free to diffuse.

To complete the surface monolayer formation, a final cycle with 2 further peptides introduced was performed (Figure 6c). At the end of the procedure, the system had been simulated under NVT for 750 ns, and 9 of the possible 15 peptides had adsorbed to the oxygen-rich silica surface.

## 4. Summary and Conclusions

In this work, we have presented a simple approach to simulating the growth of peptide films on inorganic materials. The simulation starts with an array of frozen peptides in solution above the material surface, which are then released sequentially and allowed to diffuse and adsorb. The advantages of this strategy include:

- A straightforward procedure to transition from one stage of the simulation to the next, since the atom positions are retained between stages;
- The possibility of varying how often and how many peptides are released into the system;
- Its versatility, so that it can be used with different materials, biomolecules, potentials, and software packages.

The advantages are apparent when one tries an alternative approach of placing peptides one-by-one above the surface and restarting the trajectory each time with new cycles of hydration and the addition of ions. This approach proves rather cumbersome and is more prone to human error. Of course, the method we propose does have its artefacts, in that the diffusing peptides can interact in an artificial way with frozen peptides, but we have found that once these are released the peptide pair will soon behave in a more natural way and both can adsorb to the surface. Therefore, these artefacts are short-lived and self-correcting.

Following this approach, we performed a 450 ns trajectory during which six GnRH-I peptides adsorbed to the model silica surface. The initial adlayer formed was sufficiently sparse for us to attempt to load more peptides onto it, which we performed by repeating the method with first four and then two further peptides placed frozen above the surface. By end of the 750 ns trajectory, nine peptides had adsorbed to the model oxygen-rich silica surface that is of experimental relevance. The remaining six peptides diffused away to interact with the artificial silicon-rich surface at the opposite side of the simulation cell. This behaviour suggests that the adlayer was nearing completion, and that further peptide adsorption was unlikely. Therefore, these simulations indicate that the saturated surface supports a peptide density of one per 7.6 nm^2^, a prediction that can be explored experimentally in future work.

Table 5 summarises the dominant GnRH-I configurations in the final adsorbed state, indicating also whether the dipole moment is oriented towards, away from, or parallel to the surface. The analysis of the formation of the adlayer showed that the GnRH-I can adsorb strongly to the negatively charged model silica surface, facilitated by its positively charged arginine sidechain and its N-terminus, with the negatively charge glutamic acid sidechain and C-terminus directed towards the solution. Indeed, this appears to be the dominant orientation of the adsorbed peptide, with strong adsorption of Arg8 being the consistent feature occurring in 100% of the adsorbed peptides. There was simultaneous N-terminus adsorption in 78% of these, while all had the C-terminus interacting with the solution. When strongly adsorbed, the peptides were not mobile on the timescale of these simulations. Prior to adsorption, the peptides were seen to diffuse in solution and predominantly adsorb to open spaces in the adlayer. When inter-peptide interactions occurred, lateral mobility across the surface was observed, halting when the peptide adopted a strongly adsorbed configuration.

The final structure of the adlayer is fairly sparse, with the peptides collapsed to the surface and largely free to interact with additional solutes. This concurs with our previous conclusions from simulating GnRH-I islands on the silica surface, where the orientation with bound Arg8 was important to the understanding of the drug effect of the nanoparticle-GnRH-1 conjugates. Hence our interpretation of the GNRH-I/SiNP system remains unaltered.

In conclusion, we believe that our approach to simulating peptide adlayer formation can usefully be applied to other systems, enabling the design of new vaccines and technologies. It will also be interesting to see how the growth of the peptide adlayer compares between systems, so that general design rules might emerge that accelerate the development of new applications.

## Figures and Tables

**Table 1:** Peptide colour scheme used in Figures 2–6.

Peptide Number	Colour
1	purple
2	blue
3	red
4	orange
5	green
6	cyan
7	pink
8	grey
9	brown

**Table 2:** Mean, range and standard deviation (all in Å) of the peptides’ COMs, measured for 100 ns after each peptide adsorbed.

Peptide	Mean	Range	Standard Deviation
1	6.60	1.42	0.35
2	6.46	1.63	0.39
3	7.70	1.44	0.33
4	6.77	2.89	0.70
5	6.31	1.14	0.26
6	6.47	2.50	0.46
7	8.05	4.48	1.00
8	8.79	2.00	0.32
9	10.52	0.97	0.18

**Table 3:** Peptide amino acids in contact with the silica surface at the end of the adsorption simulation. The percentage of each peptide’s amino acids in contact with the surface is shown in the final column.

Peptide	Glu	His	Trp	Ser	Tyr	Gly	Leu	Arg	Pro	Gly	%
1	✓	✓			✓	✓	✓	✓			60
2	✓	✓			✓	✓	✓	✓			60
3	✓	✓						✓			30
4	✓	✓		✓	✓	✓	✓	✓			70
5	✓	✓			✓	✓	✓	✓			60
6	✓	✓	✓				✓	✓			50
7					✓		✓	✓			30
8						✓	✓	✓			30
9	✓	✓		✓		✓		✓	✓		60

**Table 4:** GnRH-I dipole moments at various stages of adsorption: 0 = starting position; D1/D2 = diffusion moments in solution pre-adsorption; AD = at time of adsorption; 10 / 20 = 10 / 20 ns post-adsorption. The arrows signify the direction of the dipole moment vector, with dashes (horizontal lines) representing vectors parallel to the silica surface facing away from the point of view, and circles representing vectors parallel to the silica surface facing towards the point of view. The numbers show the magnitude of the dipole moments measured in D. The *x*-axis runs across the page, the *y*-axis runs normal to the page, and the *z*-axis runs up the page.

Peptide	0	D1	D2	AD	10	20
1	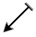	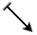	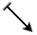	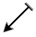	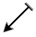	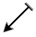
	25.47	59.36	65.39	69.40	77.41	70.02
2	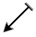	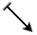	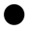	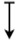	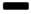	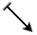
	25.47	78.78	44.94	42.79	11.84	35.19
3	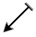	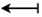	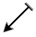	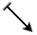	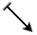	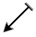
	25.47	77.27	66.77	23.97	73.23	72.59
4	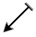	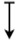	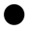	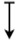	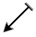	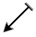
	25.47	75.51	34.84	80.75	69.34	70.09
5	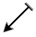	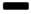	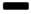	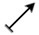	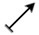	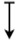
	25.47	25.52	23.96	42.57	26.17	46.52
6	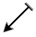	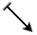	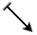	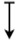	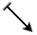	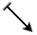
	25.47	43.38	54.39	32.37	71.01	66.76
7	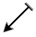	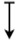	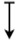	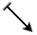	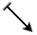	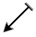
	25.47	72.87	43.79	83.70	69.56	55.52
8	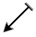	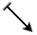	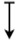	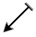	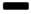	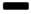
	25.47	44.65	79.98	55.24	20.19	18.35
9	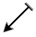	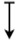	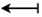	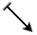	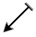	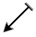
	25.47	81.46	42.95	52.66	45.22	53.15

**Table 5:** Summary of GnRH-I adsorption configurations. Dipole arrows indicate their orientation, with the down facing arrow showing dipoles facing the silica surface, the upright facing arrow showing dipoles facing away from the silica surface and the horizontal arrow shows dipoles horizontal to the silica surface.

Binding at Arg8	100%					
Binding at N-terminus	78%					
Binding at C-terminus	0%					
Orientation of dipole		89%		0%		11%
	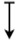		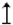		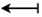	

## Data Availability

The data presented in this study are openly available in The University of Strathclyde PURE data repository at [10.15129/58a3eedc-7240-46dc-9fd8-c4f02167841f].
